# Distributions of susceptibility loci to late onset Alzheimer's disease on human chromosomes

**DOI:** 10.17179/excli2016-161

**Published:** 2016-06-22

**Authors:** Mostafa Saadat

**Affiliations:** 1 Department of Biology, College of Sciences, Shiraz University, Shiraz 71467-13565, Iran

## ⁯

Dear Editor,

Alzheimer’s disease is a major public health problem in the world. Alzheimer’s disease is a progressive, complex and heterogeneous neurodegenerative disorder. There are two types of Alzheimer’s disease: familial Alzheimer’s disease (FAD, also known as early onset Alzheimer’s disease) and sporadic type (also known as late onset Alzheimer’s disease; LOAD). FAD is relatively rare, accounting for less than 5 % of the total Alzheimer’s disease burden which is expressed as a Mendelian trait with dominant inheritance (Acosta-Baena et al., 2011[1]). In contrast to FAD, the LOAD is etiologically heterogeneous and results from a combination of many genetic and environmental factors (Gatz et al., 2006[3]). Family, twin and adoption studies have provided major evidence for the role of genetics in LOAD (Shih et al., 2004[15]). Heritability of LOAD was estimated to be up to 79 % based on twin and family studies (Gatz et al., 2006[3]).

Many single nucleotide polymorphisms have been identified and confirmed to be associated with susceptibility to LOAD (Bertram et al., 2007[2]; Rosenthal and Kamboh, 2014[10]). Several meta-analyses based on genetic polymorphisms have been widely performed to assess the association between particular gene variants and risk of LOAD (Bertram et al., 2007[2]; Rosenthal and Kamboh, 2014[10]). Some of these studies indicated that polymorphisms were not associated with the risk of LOAD (Bertram et al., 2007[2]).

Based on several lines of evidence it has been suggested that genes are distributed non-randomly on human chromosomes (Hecht, 1988[4]; Lima-de-Faria et al., 1991[6]; Mouchiroud et al., 1991[7]; Saccone et al., 1996[13]; Musio et al., 2002[8]; Rafiee et al., 2008[9]). Recently, we reported that polymorphic loci associated with susceptibility to breast cancer (Saify and Saadat, 2012[14]), schizophrenia (Saadat, 2013[11]), Parkinson's disease and multiple sclerosis (Saadat, 2014[12]) are non-randomly distributed on human chromosome segments. Taken together, we suggested that loci associated with the risk of LOAD may be distributed non-randomly on human chromosomes. Therefore the present study was carried out.

Meta-analysis studies have been published with information of polymorphisms and susceptibility to LOAD was identified using Alzheimer’s Disease Research Forum (AlzGene database) (http://www.alzgene.org) electronic database (Bertram et al., 2007[2]) and from the study of Rosenthal and Kamboh, 2014[10].

There were 1395 studies concerning the associations between 695 genes (2973 polymorphisms) and risk of LOAD. Table 1(Tab. 1) shows the genes which their single nucleotide genetic polymorphisms associated with LOAD susceptibility in at least one ethnic group. There are 54 loci associated with the risk of LOAD. The method of Tai et al. (1993[16]) was used to evaluate the non-randomness distribution of susceptible loci on each chromosomal band(s). The relative width of each band was measured using the diagram of the International System for Chromosome Nomenclature (ISCN, 1981[5]). A probability of P < 0.05 was considered statistically significant. 

Statistical analysis revealed that the LOAD susceptible loci distributed non-randomly on human chromosomes. Human chromosome segments 19q13 (P < 0.001) and 6p21.1 (P < 0.001) were bearing significantly higher numbers of susceptible loci for LOAD. There are nine and three genes which associated with susceptibility to LOAD on 19q13 (*GAPDHS, PLD3, BCAM*,* PVRL2*, *TOMM40*, *APOE*, *APOC1*, *APOC4*, and *CD33*) and 6p21.1 (*TNF*, *HLA-DRB5*, and *TREM2*) chromosome segments, respectively.

The present study revealed that the human chromosome segments 19q13 and 6p21.1 were bearing significantly higher numbers of susceptible loci for developing late onset Alzheimer’s disease. The present finding has two important aspects: First, genes did not randomly distribute on human chromosomes. Second, as mentioned previously for other multifactorial traits (Saify and Saadat, 2012[14]; Saadat, 2013[11], 2014[12]), a mass screening test might be designed using polymorphic loci located on these chromosome segments (particularly for the segment 19q13) for diagnosis of LOAD.

Previously it has been reported that polymorphic loci associated with risks of breast cancer (Saify and Saadat, 2012[14]), schizophrenia (Saadat, 2013[11]) and multiple sclerosis (Saadat, 2014[12]) non-randomly located on human chromosome segments 19q13, 6p21, and 19q13, respectively. It could be concluded that polymorphic loci on a particular chromosome segment have significant associations with different diseases. It should be noted that at present it is impossible to explain the biological significance of the defined clustering of genes in the etiology of the LOAD or other multi-factorial disease. 

## Acknowledgements

This study was supported by Shiraz University.

## Conflict of interest

The authors declare no conflict of interest.

## Figures and Tables

**Table 1:**
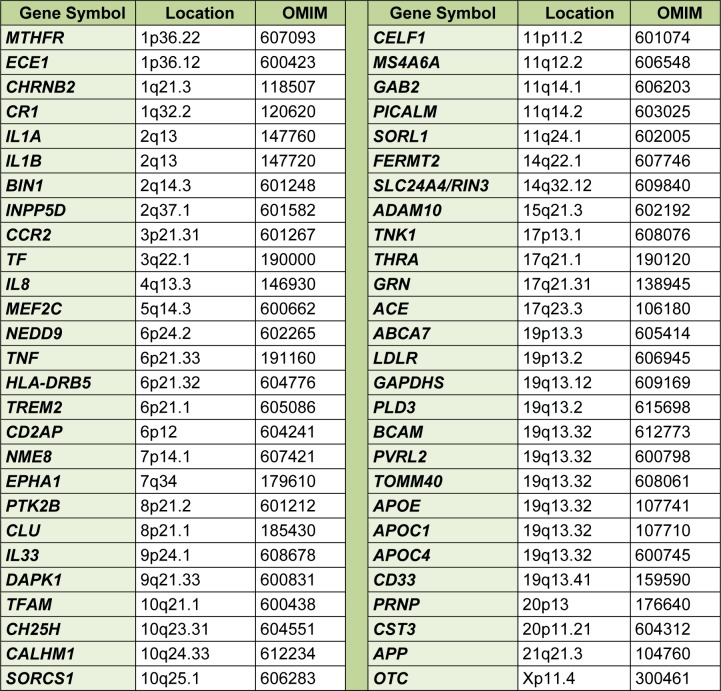
List of polymorphic genes associated with susceptibility to late onset Alzheimer's disease
